# Profiling the impact of the embryonic microenvironment through global transcriptomic and DNA methylome surveying

**DOI:** 10.1186/1756-8935-6-S1-P76

**Published:** 2013-03-18

**Authors:** Habib A Shoiaei Saadi, Dominic Gagné, Eric Fournier, Isabelle Laflamme, Marc-André Sirard, Claude Robert

**Affiliations:** 1 Centre de recherche en biologie de la reproduction, Université Laval, Quebec City, QC, G1V0A6, Canada

## 

Transcriptomicanalyses have been conducted to study how mammalian early embryos cope with their immediate microenvironments especially when stressed from the application of assisted reproductive technologies, maternal nutrition or any other induced external factors. To study potential long term impacts from these stresses, description of early embryo epigenome is necessary. DNA methylation profiling of mammalian early embryo presents important challenges as they are rare biological material. Very recently, a genome-scale, base-resolution timeline of DNA methylation has been characterized for mouse embryo[1], however, such information has not been reported for treated embryos or for other species. We focus on bovine embryonic development since it follows a similar kinetic and developmental success rate as human. Using our transcriptomic platform[2] studies revealed that several key pathways seems to react when embryos are submitted to stress but it was also observed that long non-coding RNAs (IncRNAs) represent the class of RNA that are the most influenced by the embryonic environment further supporting the involvement of epigenetic mechanisms in the embryonic response.

Commercial platform specific to the study of the bovine methylome do not exist yet. We therefore set to develop one with the constraints of enabling to survey the general topology of the genomic methylome using the limited amount of starting material offered by early embryos and also to provide a cost effective platform with a complete data analysis pipeline. We report the development of the Bovine Embryonic Global Methylome platform (BEGMP) which allows global DNA methylation from as little as 7-8 ng of gDNA starting material (representing 10 expanded blastocysts). Samples are processed through a modified HpaII Tiny Fragments (HTFs) enrichment by ligation-mediated PCR (HELP) assay. Initial surveying of the methylome found in Day-7 and Day-12 bovine embryos suggests a transition during development of methylation marks in repeat containing elements in a class-specific manner showing the potential of the platform to describe the ontology of the establishment of methylation marks during early embryogenesis. The platform was also used to study the impact of embryonic culture conditions where both the transcriptome and the methylome were surveyed from the same samples. Treatment of *in vitro* cultured embryos in presence of the methtyl donor S-adenosyl methionin showed that in addition to protein coding genes, IncRNAs were also impacted by the treatment. At the DNA methylation level, it showed global hypermethylation in treated embryos and parallel analysis revealed some similar function and network between early embryo transcriptome and methylome. Overall, BEGMP is the first developed and functional methylome platform for non-model spices which allows to survey the transcriptome and methylome from very restricted samples.
